# Optimized Placement of Frost-Measuring Sensors in Heat Exchangers via Image Processing of Frost Formation Pattern

**DOI:** 10.3390/s23115253

**Published:** 2023-06-01

**Authors:** Martim Aguiar, Pedro Dinis Gaspar, Pedro Dinho Silva

**Affiliations:** 1 Faculty of Engineering, Department of Electromechanical Engineering, University of Beira Interior, Rua Marquês d’Ávila e Bolama, 6201-001 Covilhã, Portugal; martim.aguiar@ubi.pt (M.A.); dinis@ubi.pt (P.D.G.); 2 C-MAST-Centre for Mechanical and Aerospace Science and Technologies, 6201-001 Covilhã, Portugal

**Keywords:** heat exchangers, frost formation, frost sensing, refrigeration efficiency, computer vision

## Abstract

Heat exchangers (HXs) play a critical role in maintaining human thermal comfort and ensuring product safety and quality in various industries. However, the formation of frost on HX surfaces during cooling operations can significantly impact their performance and energy efficiency. Traditional defrosting methods primarily rely on time-based control of heaters or HX operation, overlooking the actual frost formation pattern across the surface. This pattern is influenced by ambient air conditions (humidity and temperature) and surface temperature variations. To address this issue, frost formation sensors can be strategically placed within the HX. However, the non-uniform frost pattern poses challenges in sensor placement. This study proposes an optimized sensor placement approach using computer vision and image processing techniques to analyze the frost formation pattern. Through creating a frost formation map and evaluating various sensor locations, frost detection can be optimized to control defrosting operations with higher accuracy, thereby enhancing the thermal performance and energy efficiency of HXs. The results demonstrate the effectiveness of the proposed method in accurately detecting and monitoring frost formation, providing valuable insights for sensor placement optimization. This approach presents significant potential in enhancing the overall performance and sustainability of the operation of HXs.

## 1. Introduction

**Figure 1:**
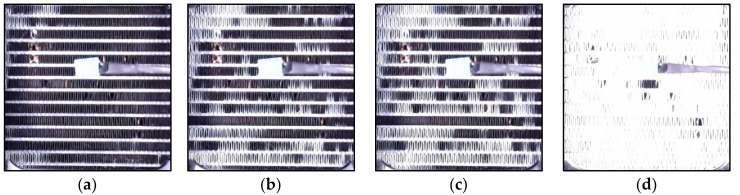
Ordered sequence illustrating the frost development process on the intake side of a heat exchanger: starting from slight accumulation (**a**), transitioning through moderate (**b**) and substantial (**c**) frost build-up, culminating in total blockage (**d**) [9].

Frost formation remains a significant challenge to the efficiency of air conditioning and refrigeration systems. In light commercial systems, heat exchangers (HXs) with a large area-to-volume ratio are particularly susceptible to frost accumulation on fin surfaces under subfreezing temperature conditions [1,2,3]. The presence of frost acts as a thermal barrier, reducing thermal performance and restraining heat transfer between air and refrigerant [4,5], besides increasing the energy consumption [6,7,8]. The influence of frost should be taken into account during the design and dimensioning of the HX, as without an appropriate design and defrosting method, the buildup of frost restricts or even blocks airflow between fins, resulting in increased energy consumption, lower performance, degradation of refrigeration conditions, decreased product safety and quality, or even system damage [9,10]. Figure 1 provides a visual representation of the frost formation process in a heat exchanger. It shows a sequential sequence of images, labeled (a) to (d), illustrating the gradual buildup of frost on the intake side of the heat exchanger.

Various defrosting methods have been developed to address frost formation [11]; however, it is important to note that these methods might require additional energy consumption for their operation [12]. Frost inhibition methods are known as restraint frost, while those that remove formed frost are called frost removal [13]. Implementing defrosting methods in industrial systems can be complicated by factors such as cost, complexity, and unreliable prediction and sensing methods. Timed defrost operations, still widely used, must be configured to accommodate the worst-case scenario without considering the variations in parameters that affect frost formation. These fluctuations result in different levels of frost formation throughout the day and season, requiring varying defrost cycle durations [14].

Demand defrost aims to address the frost formation issue through predicting its occurrence via the calculation of influencing parameters, detection of accumulation symptoms, or direct frost measurement [15]. Various sensing methods have been investigated to directly measure frost formation, including capacitive [16]; photoelectric [17]; piezoelectric [18]; optic fiber [19]; resistive sensors, as emphasized in previous works [20]; and others [21].

**Figure 2:**
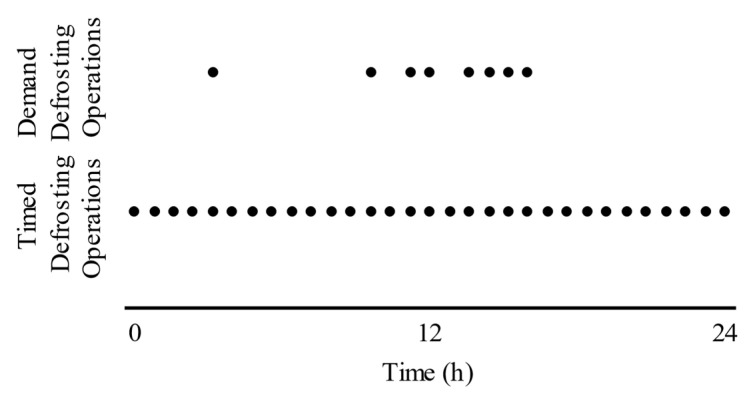
Comparison between timed and demand defrosting using the TEPS sensor, adapted from [14].

Ge et al. [14] developed the Tube Encircled Photoelectric Sensor (TEPS) and conducted experiments to evaluate its performance in comparison to timed defrosts in HXs. The results of their study demonstrated the potential of using a demand defrost method in significantly reducing the frequency of defrosting operations. In a specific case presented in Figure 2, which spanned a 24 h period, the TEPS sensor required only 8 defrosting operations compared to the 30 defrosts scheduled for the worst-case scenario based on timed defrosts. Importantly, these eight defrosting operations were not evenly distributed throughout the day, but rather as required according to the amount of frost accumulated on the HX, resulting from different conditions throughout the day. This approach of adaptive defrosting resulted in improved energy efficiency and minimized the additional thermal loads imposed by frequent and unnecessary defrosting operations. The findings highlight the potential of frost sensing for optimizing the defrosting process in HXs, leading to enhanced overall system efficiency and performance.

To assess the accuracy of these sensors, an accurate frost detection method must be employed to cross-check sensor measurements with actual frost formation.

Computer vision techniques have been employed to detect frost formation in HXs [22,23]. These methods use image processing algorithms to analyze images of the HX surface and identify areas of frost formation [24]. This approach has shown promising results in accurately detecting frost formation and can potentially provide a more efficient and reliable method of controlling defrosting operations such as detecting the ideal time to perform a defrosting operation [24].

In a mainstream application, the use of computer vision methods for frost detection in the HX of refrigeration systems can provide accurate results, but the need for specialized equipment, complex setups, and processing power can present challenges in terms of cost, space, and practicality. A small low-cost sensor, such as those developed in previous works [20], could provide a robust alternative that can achieve sufficient accuracy to be effective in providing data to control demand defrosting operations. In this case, computer vision could have a role in the implementation study of these sensors.

Prior research [9] developed a computer vision method for measuring frost formation to corroborate data obtained from a resistive sensor. However, as anticipated, the results indicated that frost formation was uneven across the HX, and sensor placement produced very specific results based on its position. Conversely, the computer vision method assessed the average frost across the HX, without considering the frost formation pattern or the particular sensor placement within the HX.

This study introduces a novel application of computer vision in analyzing frost formation in HXs for the optimization of sensor placement. Unlike previous approaches that primarily focused on using computer vision for frost detection or to control defrosting operations, our research explores the usage of computer vision as a tool for the optimization of the placement of frost-measuring sensors. This method is an improvement on previous works [9]. Through dividing the HX image into smaller sections, our approach provides valuable insights into the distribution and patterns of frost formation within the HX, facilitating the optimization of sensor placement. This use of computer vision allows for a more efficient demand defrost system, as it enhances sensor placement, therefore improving sensor accuracy. The collected data are combined to generate a comprehensive frost formation map, enabling an in-depth analysis of highly susceptible areas prone to frost buildup.

## 2. Materials and Methods

**Figure 3:**
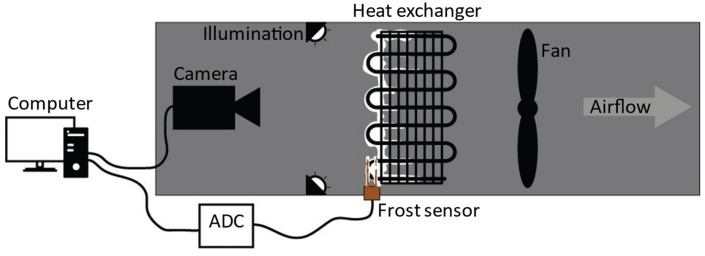
Diagram of the experimental arrangement.

The experimental setup involves an HX located in a tunnel with air flow forced through a fan. The HX is cooled using a refrigerant below the freezing point of water. When the air encounters the cooled HX surface, below the dew point, water condenses and forms frost. This setup is illustrated in Figure 3.

A resistive sensor was utilized to detect moisture, frost, or a dry surface within the HX through absorbing condensation. A camera was placed directly above the center of the HX at a perpendicular and level angle to minimize image distortion. Additionally, a fixed light source was aimed at the HX to eliminate any noise resulting from external lighting fluctuations. Two crucial aspects of the experiment setup require emphasis: firstly, the sensor used to detect frost formation and the experimental design utilized for conducting the tests, and secondly, the steps involved in capturing the images and analyzing the resultant data.

### 2.1. Sensor for Frost Detection

In previous studies [20], a low-cost resistive sensor for detecting water accumulation and frost formation in an HX was developed. The sensor is inexpensive, compact, and straightforward, comprising two 0.5 mm copper electrodes that are linked and insulated by a cotton string serving as both an electrical insulator and a moisture absorbent. The voltage drop across the sensor’s terminals varies based on the material that has accumulated between the electrodes (air, water, or ice) due to differences in electrical resistivity. Consequently, a correlation can be established between the materials. If the sensor is placed between the HX fins with its electrodes positioned closely enough to measure a voltage drop when voltage is applied, a characteristic voltage drop will be measured as water accumulates. The voltage drop will increase as the water freezes and, subsequently, evaporates during defrost. Previous studies [25,26,27,28,29] have utilized this characteristic in the development of a device. The resistance of the sensor and, consequently, the voltage drop vary according to Equation (1).

R = ρ × L/A(1)

**Figure 4:**
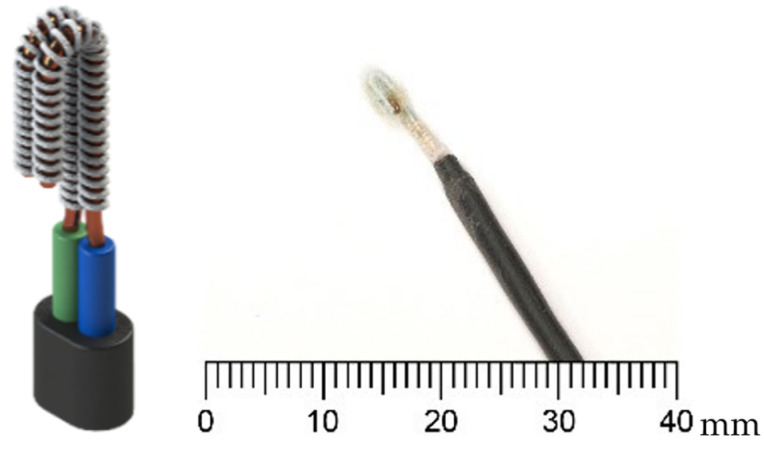
Sensor CAD model (left) and photo (right), as described in [15].

The sensor’s overall resistance, denoted by R [Ω], experiences fluctuations based on the resistivity ρ [Ω.m] of the material between the electrodes. This variance arises due to the dissimilar electrical resistivity values of air, water, and ice. L [m] represents the length of the material or the distance between the electrodes, while A [m^2^] represents the cross-sectional area of the connection between the electrodes. As water condenses on the HX surface, it is assimilated by the cotton string of the sensor, causing a decrease in fabric resistivity, and resulting in a reduction in the voltage drop across the sensor terminals. Conversely, when frost is formed, resistivity increases, leading to a rise in the voltage drop, which is then detected as frost. During the defrosting process, water is once again detected until the HX is dry. The sensor, depicted in Figure 4, is clamped to one of the HX fins.

The voltage drop between the electrodes is gauged using an 8-bit analog-to-digital converter (ADC) linked via a voltage divider. The ADC provides a reading ranging from 0 to 1023, which can be transformed into voltage drop. However, to simplify analysis and comparison, the reading is left unconverted.

### 2.2. Method for Image Acquisition and Processing

**Figure 5:**
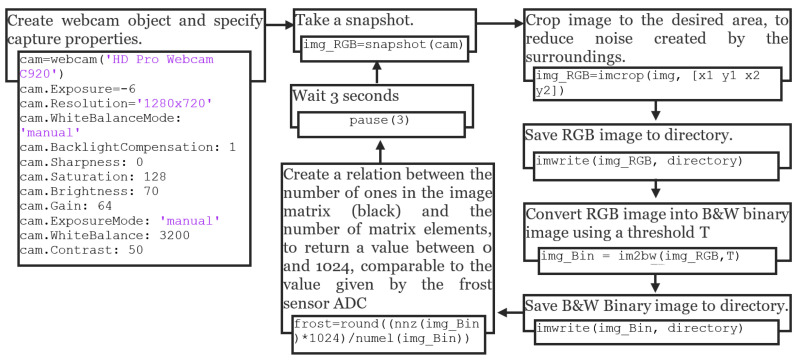
MATLAB logic for analyzing overall frost formation average, as per [8].

To ensure the veracity of the sensor measurements, a Logitech C920 webcam was placed in front of the HX, and MATLAB version R2018b was employed for capturing and processing images using the approach illustrated in the diagram depicted in Figure 5.

**Figure 6:**
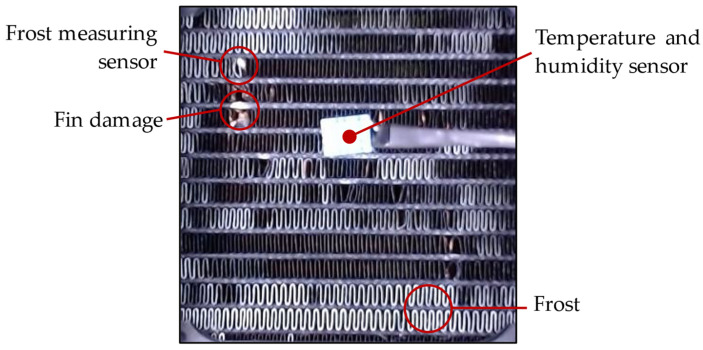
Annotations on the HX image highlighting sensor position and key features to consider, adapted from [8].

The process of capturing and analyzing images of the HX commences with MATLAB creating a webcam object and defining fixed capture properties to maintain uniformity. Illumination is provided via two 12 V DC white LED strips with 9 SMD 5050 LEDs each to assure constant lighting conditions. A static image is then captured and cropped to display solely the HX, as illustrated in Figure 6.

Following the capture of a static image of the HX, the image is converted into a black and white (B&W) binary image using a threshold. The threshold was calibrated so that a fully frosted HX appears predominantly white, while a completely dry HX appears almost entirely black. Notably, a binary image differs from a grayscale image in that each pixel in a grayscale image ranges in value from 0 to 255, while each pixel in a binary image has only a value of 1 or 0. For the current camera settings and illumination parameters, the threshold is set to 50%. This means that a gray pixel with a value between 0 and 127 is considered 0, which translates to black, and a gray pixel with a value between 129 and 255 is considered 1, which translates to white. Subsequently, the number of black pixels is tallied, and the ratio of black to white pixels is calculated to fall between 0 (fully black) and 1023 (fully white), thereby ensuring that the camera-based frost measurements fall within the same range as those measured with the sensor’s ADC.

**Figure 7:**
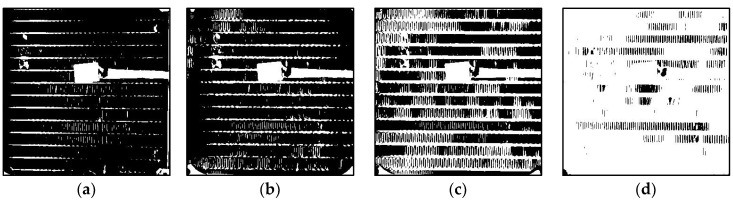
Stages of frost formation on the HX depicted as black and white binary images, ordered from slight accumulation (**a**), transitioning through moderate (**b**) and substantial (**c**) frost build-up, culminating in total blockage (**d**).

To accommodate the rapid frost and defrost cycles characteristic of the experimental setup, an image sample is captured, processed, and saved in RGB and B&W binary formats every three seconds. Although a three-second data acquisition rate may be considered high, it provides sufficient time to collect and process the data effectively, allowing for comprehensive analysis of dynamic phenomena, including the faster transitions observed during defrosting processes, such as frost melting. Figure 7 displays the results of three distinct stages of frost formation. While the temperature and humidity sensor or damage to the fins in the HX may introduce some noise in the readings, these values remain constant and have a negligible effect on the readings.

**Figure 8:**
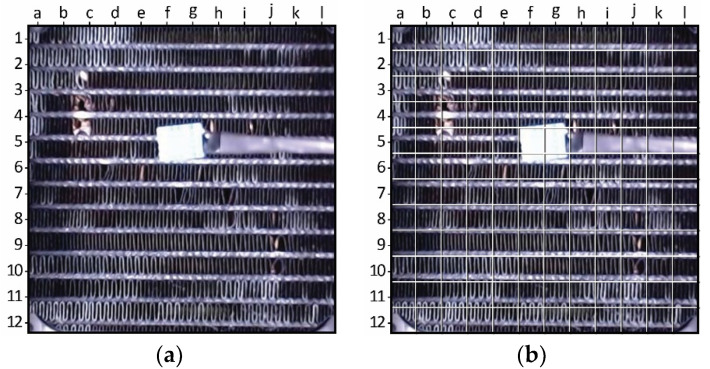
Image of the HX, both without (**a**) and with (**b**) a grid overlay that delineates the divided slices.

To gain a more comprehensive understanding of the frost formation in different regions of the HX, it was essential to divide it into smaller sections encompassing both the fins and refrigerant tubing. This is particularly crucial since the tubing is among the first parts of the HX to experience frost formation. Through creating 144 tiles that include both fins and refrigerant tubing, the HX is divided into 12 × 12 sections, ensuring that the resulting map provides an accurate representation of frost formation. This prevents any biases that may arise from irregular slicing, which could result in either the tubing or the fins being missed. Figure 8 displays a photograph of the HX, including a superimposed grid that delineates the HX into tiles. The grid features designated labels for the horizontal and vertical tiles to assist in the analysis of results.

**Figure 9:**
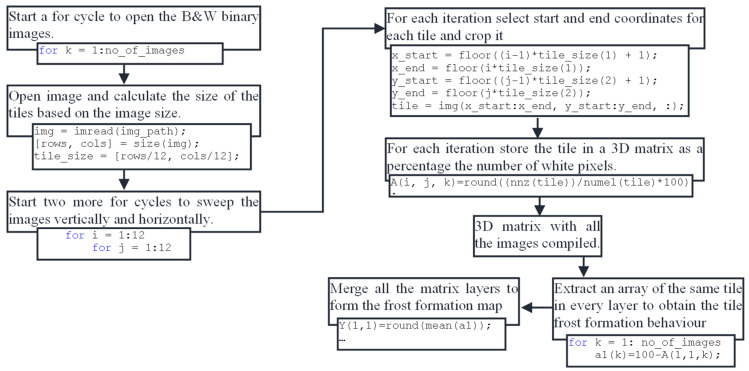
MATLAB logic used to analyze tile frost formation and the resulting frost formation map.

This study utilizes specific grid labels shown in Figure 8 to refer to individual tiles, such as “a1” for the top left corner or f5 and g5 for the tiles likely to yield poor results because of their coverage by the temperature and humidity sensor. While the colored image in Figure 8 provides better visualization and representation of the HX, black and white binary images are employed for the actual computing of the tiles. The logic behind the image processing of the tiles is represented in Figure 9.

**Figure 10:**
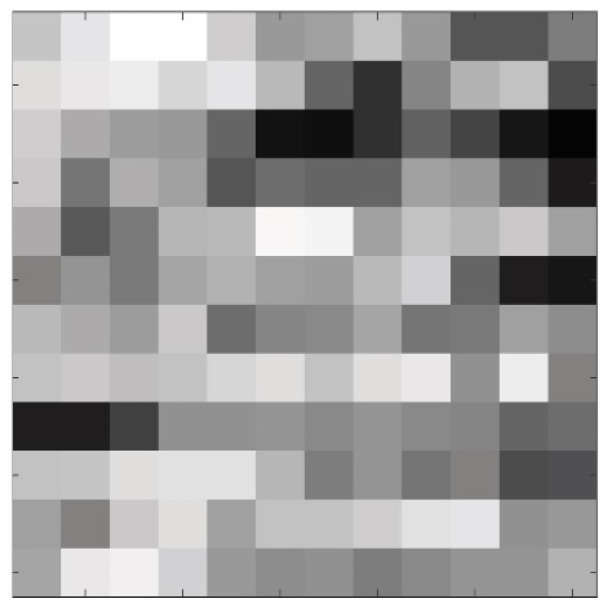
Representation of frost formation on a single layer of the 3D matrix.

The black and white binary image is loaded into MATLAB, and the program determines the image’s division based on its resolution, resulting in the creation of 144 square tiles. For each tile, the number of white pixels, representing frost, is counted, and the percentage of white pixels is recorded in a three-dimensional matrix. This 3D matrix comprises 12 × 12 2D matrices, with each element representing the percentage of frost in its corresponding tile. Figure 10 displays a single layer of the 3D matrix that represents the frost formation at a particular time, providing a visual representation of the distribution and intensity of frost formation in different regions of the HX.

**Figure 11:**
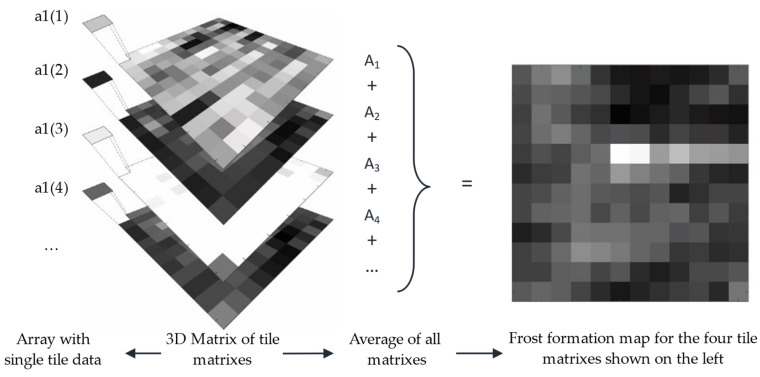
Graphical representation of how data are extracted from the 3D matrix of frost formation.

Two types of information can be extracted from the 3D matrix, which is better illustrated in Figure 11. Firstly, it is possible to observe the fluctuations in frost formation in a specific tile over time through taking an array with the same row and column elements from each layer. The resulting data can be plotted to provide more detailed information about localized frost formation, as will be demonstrated in the results section. Secondly, the layers can be merged into a 2D matrix that represents the average of all the tiles, allowing for an analysis of the overall pattern of frost formation. This information can be used to optimize the positioning of frost detection sensors or design the defrosting system while taking the frost formation pattern into account.

Following the execution of the experiments using the methods described in this section, the results of the study are presented herein. In the following section, the findings will be analyzed, and the collected data will be interpreted. The aim is to identify any patterns and trends in the data and highlight any limitations or challenges encountered during the experimental process.

## 3. Results

**Figure 12:**
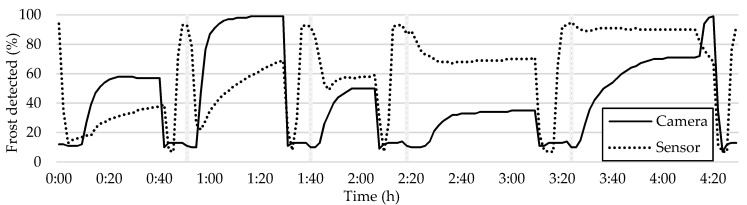
Overall average frost formation and sensor readings.

Prior to presenting the results of the current study, it is important to examine the outcomes of previous research. Figure 12 depicts a comparison between the graph of the average frost formation and the plot of the sensor readings.

In order to compare the sensor readings to the camera readings, it is essential to interpret the sensor data. The sensor initially detects air, which has high resistivity, and as moisture accumulates, the resistivity decreases. When the water freezes, the resistivity increases, and the sensor detects the frost. Upon initiation of a defrosting cycle, the frost melts, and the water’s resistivity decreases again until the water dries, and the resistivity increases, marking the end of the defrosting cycle and the start of a new cycle (as represented by the vertical bars in Figure 12). Despite some fluctuations, the sensor measures five frost–defrost cycles accurately, which is further validated via the camera readings.

**Figure 13:**
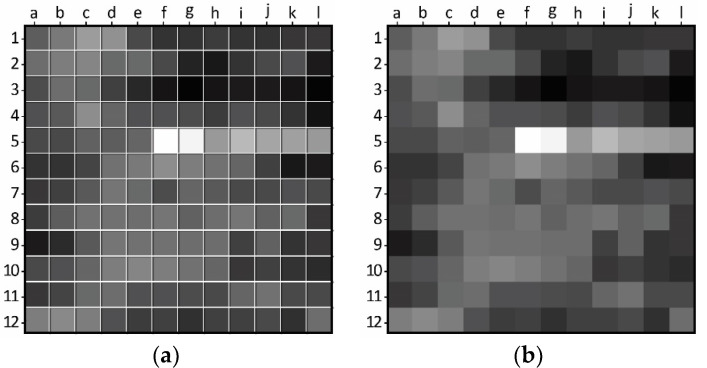
Frost formation map comprised of labeled tiles, using numerical references 1-12 on the vertical axis and alphabetical references a-l on the horizontal axis, shown with (**a**) and without (**b**) tile demarcation.

Before analyzing the variations in frost detection across different tiles, it is relevant to examine the frost formation map to identify the most interesting tiles to focus on. Figure 13 illustrates the frost formation map derived from the experimental results and can be studied to select the tiles for closer examination.

Based on the frost formation map presented in Figure 13, it is evident that certain regions of the HX have a greater accumulation of frost. However, this visualization also exposes an artifact resulting from the temperature and humidity sensor (from f5 to l5) as well as spots where the HX is damaged. A careful analysis of Figure 8 was conducted, and tiles were selected for further examination, with an emphasis on different research objectives, as outlined in Table 1.

**Figure 14:**
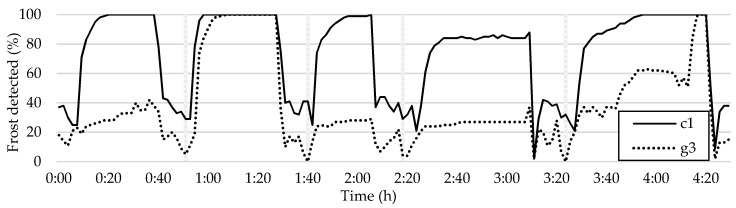
Comparison of frost detection between two randomly selected tiles with different readings.

Upon analyzing two tiles arbitrarily, it becomes evident that the sensitivity of frost detection can vary depending on the tile’s location. This observation is critical, as it highlights how the frost detection method can improve accuracy while reducing its effectiveness. Figure 14 illustrates that a reading from tile c1 can be much more precise than the overall image average, but it also shows how the readings from tile g3 struggle to detect frost formation, except for the second frost–defrost cycle.

**Figure 15:**
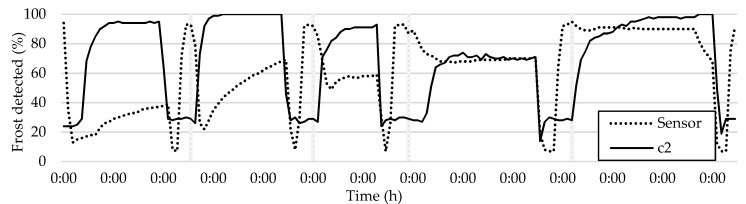
Comparison of sensor readings and frost formation in tile c2, where the sensor is located.

Two undamaged tiles, c1 and g3, with different brightness in the frost formation map, were analyzed for their sensitivity to frost detection. The analysis indicated that areas that appear brighter in the frost formation map are likely to have a higher sensitivity to frost detection. Tile c2, where the frost measurement sensor was primarily placed, is expected to provide more accurate results compared to the sensor readings, assuming the sensor is functioning properly. The comparison between tile c2 and the sensor readings is presented in Figure 15.

**Figure 16:**
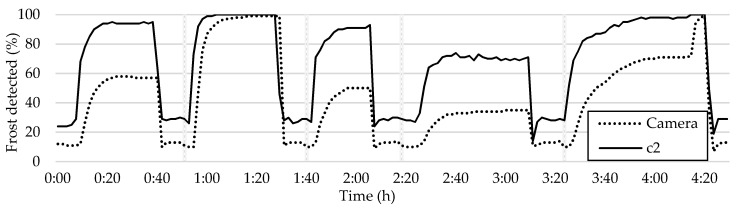
Comparison between the frost detection in c2 and the overall average.

The analysis of frost in tile c2 highlights the effectiveness of this method in verifying frost sensing measurements. However, it is crucial to note that the method has limitations in differentiating between a wet and dry HX, which results in the graphs showing the resistive sensor’s detection of water drying. Additionally, the tile lags behind the sensor readings in detecting frost, especially in the case of black ice, which is virtually invisible to the camera. Despite this, the tile detects frost before the overall average method in all the frost–defrost cycles, proving to be a superior method, as demonstrated in Figure 16.

Analyzing the frost formation in tile c2 has demonstrated the method’s effectiveness in verifying frost sensing measurements. However, it is important to acknowledge the method’s limitations in distinguishing between a wet and dry HX, as the resistive sensor’s detection of water drying is shown in the graphs. Furthermore, the tile’s ability to detect frost lags behind the sensor readings, particularly in the case of black ice, which is difficult to detect with the camera. Nonetheless, the tile detects frost before the overall average method in all the frost–defrost cycles, indicating its superiority as demonstrated in Figure 16.

The method of detecting frost using individual tiles results in more irregular curves due to the smaller sample size, as opposed to the overall average method which produces smoother curves.

In the current study, the tiles represent only 1/144th of the original image area, which accentuates the irregularities in frost formation within each tile. Smaller tiles will result in more irregular curves, while larger tiles will yield smoother curves but may compromise the accuracy of frost detection in a specific area. It is essential to strike a balance between tile size and computational power to obtain accurate and representative results, especially for real-time monitoring.

**Figure 17:**
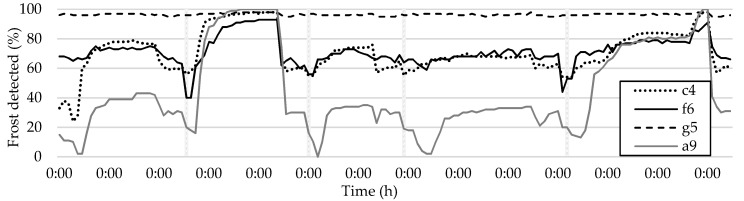
Plot of tiles with abnormalities.

Tile c2 consistently detects more frost than the overall average method, as demonstrated by the lighter color of the tile in the frost formation map. The curves from individual tiles and the frost formation map can also be used to diagnose issues in the HX, such as blockages, inefficient defrosting, poor HX performance, or damage. Unexpected behavior in certain tiles, such as c4, f6, g5, and a9, can be seen in Figure 17.

Tile c4 exhibits abnormally high values due to its location in a damaged area of the HX that reflects more light—whiter in the picture. Tile g5 is not possible to analyze due to being obscured by a white temperature and humidity sensor. Despite appearing normal, tiles f6 and a9 have suffered performance degradation due to the fins becoming disconnected from the refrigerant tubing.

## 4. Discussion

**Figure 18:**
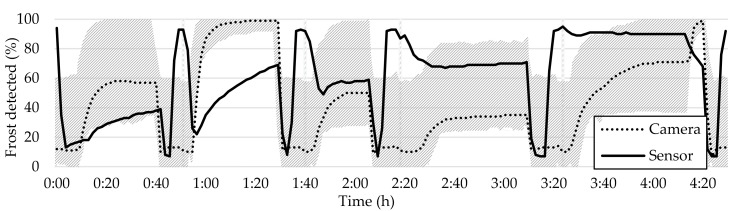
Representation of the amplitude of frost formation detection, with the shaded region indicating the range of values observed across all tiles.

The main goal of this study is to determine the best location for placing a frost detection sensor. As discussed in the previous chapter, there is significant variability in frost formation across the HX over time. Figure 18 supports this conclusion through presenting a shaded region that displays the amplitude of frost detection across tiles, along with a plot of the frost detection sensor readings and the overall camera average. The values of tiles that exhibit abnormalities were excluded from the plot.

The shaded area in Figure 18 highlights the importance of selecting the optimal location for the frost-measuring sensor. One interesting observation is that the severity and uniformity of frost accumulation in the HX can be analyzed according to the amplitude of the shaded area in Figure 18. A high amplitude shaded area indicates non-uniform frost distribution, while a narrow-shaded area indicates uniform frost distribution or absence of frost. The amplitude of the shaded area for frost–defrost cycle 5 suggests that there was a severe and uniform accumulation of frost across the HX during this cycle.

Several methods are proposed for selecting the best tile to place the sensor, given the high disparity in frost formation across the HX. Different methods may have distinct applications and should be selected accordingly.

### 4.1. Most Representative Tiles Method

**Figure 19:**
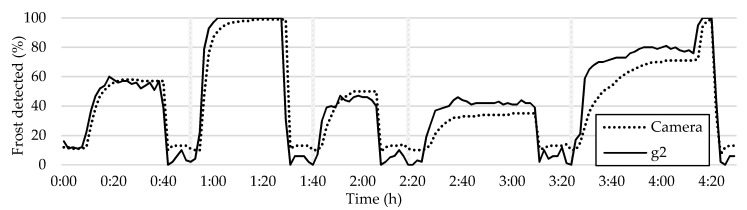
Comparison between frost formation detection in tile g2 and the HX average.

The aim of this approach is to identify the tiles that best represent the average frost formation pattern. To achieve this, all the tiles are plotted along with the camera average curve. The tiles that have curves closest to the camera average plot are then selected. An accurate representation of the average frost formation can be seen in tile g2, as shown in Figure 19.

Through comparing the frost formation curve in tile g2 to those shown in Figure 14, Figure 16 and Figure 17, it becomes evident that it accurately represents the overall frost formation with an acceptable degree of accuracy. In addition, tile g2 consistently provides an accurate representation of the frost formation across all five frost–defrost cycles. This is not the case for tile c2, as shown in Figure 16, where it is plotted against the camera average. Although all the cycles are accurately measured, there are significant differences in the detected values when compared to the overall average.

### 4.2. Early Warning Trigger Method

**Figure 20:**
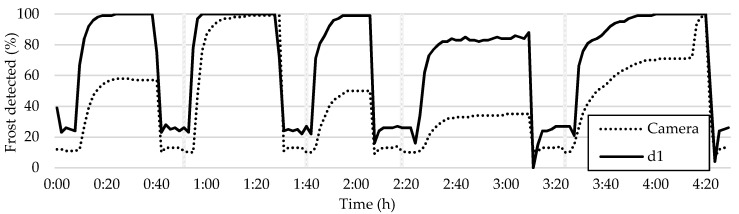
Comparison between frost formation detection in tile d1 and the HX average.

The proposed early warning trigger method suggests placing the frost detection sensor in the tiles where frost formation occurs first. These tiles are not only the most critical and prone to blockage but also allow for early detection of frost before it becomes problematic. This approach can be used to provide timely and sensitive warnings, particularly in cases where even a small amount of frost formation can significantly affect the system’s performance. To accomplish this, the frost formation map displayed in Figure 13 is consulted, and a tile with a lighter tone, such as d1, c1, b12, c12, etc., is chosen, excluding any abnormalities. To illustrate this, the plot of the highly sensitive frost detection of tile d1 is presented in Figure 20.

Frost detection in tile d1 is rapid and highly sensitive, as shown in the plot of its frost detection in Figure 20. In all the frost–defrost cycles, frost formation was detected significantly earlier in tile d1 compared to the overall average of the HX. Even during frost–defrost cycles where the plot of the average frost formation shows little to no frost detection, high values of frost detection are observed consistently in this tile. This method proves useful in detecting frost formation even in cycles where low frost formation is observed, as in cycle 4 of the analyzed case. Such early detection could be useful in preventing performance degradation in systems sensible to even small amounts of frost formation. A less sensitive method may be more appropriate for resilient systems that are only affected by frost formation when the HX is severely blocked by frost.

### 4.3. Late Warning Trigger Method

**Figure 21:**
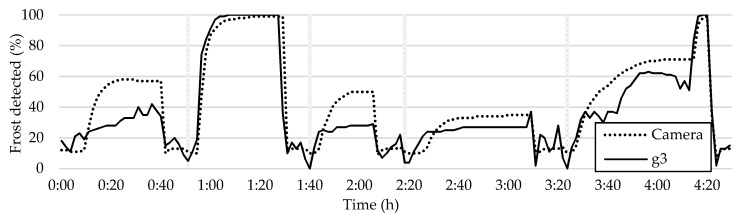
Comparison between frost formation detection in tile g3 and the HX average.

The late warning trigger method suggests monitoring the tiles where frost formation occurs last. This approach could prevent unnecessary defrosting operations, which come with performance costs and undesired thermal loads. To implement this method, one can consult the frost formation map presented in Figure 12 and select a darker tile, such as g3, i3, i6, k12, etc., while excluding abnormalities. Figure 21 shows the plot of tile g3 as an example of this method.

Upon initial inspection, the measurements may seem unreliable and inaccurate. However, this is not the case, as in cycles 1, 3, and 4, the frost accumulation was insufficient for this method of frost sensing to trigger a defrosting operation. In contrast, during frost–defrost cycle number 2 and the end of frost–defrost cycle 5, the severity of the frost accumulation was sufficient for the tile to detect the presence of frost with clarity, as evidenced by the high values of frost detection shown in the shaded area in Figure 18. These results point out that the optimal measurement method is not evident and requires careful analysis.

### 4.4. Selecting a Method

To effectively sense frost in a HX with uneven frost distribution, consideration must be given to the frost formation map and the area in which the frost sensor is placed. To compare frost formation in tiles with varying sensitivities, Table 2 presents a comparison of frost formation in tiles g2, d1, and g3.

Table 2 displays the frosting phase during frost–defrost cycle 2, highlighting the significant impact that sensor placement can have on frost detection and the crucial role of accurate measurement of the state of frost formation in the HX.

Choosing the ideal tile for placing the frost detection sensor depends on the application’s frost sensitivity requirement and the number of sensors that can be installed. A method that could result in the most precise understanding of frost formation in the HX is placing three sensors in tiles with different frost formation behaviors. The first sensor is placed in a tile with behavior similar to the average to measure overall frost formation, the second in a highly sensitive tile to provide immediate frost formation warning, and the third in a low frost formation tile to indicate a critical moment in frost formation. However, using three sensors could add complexity to the system, which should be as resilient and reliable as possible throughout many cycles. Therefore, a better application could be a single sensor placed in a tile chosen based on the information from the frost formation map and system requirements.

One possible approach with two sensors would be to use both the highly sensitive and low-sensitivity tiles and analyze the data from both to determine the optimal time for defrosting operations. Alternatively, two tiles that are representative of the average frost formation can be selected, and the data from both can be used to confirm each other, thereby increasing reliability and resilience.

Finally, if only one sensor is used, it is recommended to place it in a tile in which the sensitivity better suits the application, to increase the efficiency of the system and the stability of the temperature of the refrigerated goods. It is important to keep in mind that the chosen tile does not necessarily have to be one with extreme readings, but rather one of the most suitable results. The selection of the appropriate method is not a clear-cut decision and involves a spectrum of sensitivities.

## 5. Conclusions

The objective of this investigation was to measure frost formation across an HX using an image analysis technique in order to corroborate data measured from a resistive sensor and optimize its placement within the HX. The findings emphasized the significance of initially examining the pattern of frost formation in order to gain a better understanding of the process before installing the frost-measuring sensor.

Creating a frost formation map can serve as a crucial tool in analyzing the frost formation pattern, aiding in the placement of a frost detection sensor in a spot with ideal sensitivity for the specific application.

Furthermore, the irregular frost formation patterns in specific tiles can be utilized to identify potential issues in the HX, including blockages or damage to the fins.

The method demonstrated has the capacity to complement or enhance current frost sensing techniques.

A higher tile resolution requires more computational power to process the data, which is not an issue when developing a frost map with previously gathered data. However, it can become critical when conducting real-time measurements with high sample rates.

While the image analysis method for frost detection in HXs shows potential for improving sensor placement and reliability of defrosting operations, practical methods for obtaining a frost formation map must be developed, as each HX is unique.

This study emphasizes the intricacies of frost sensing in HX and the significance of monitoring frost formation to achieve optimal performance and energy efficiency.

The sensitivity of frost detection using this image analysis method can be adjusted through manipulating camera settings, such as exposure time, to achieve the desired level of sensitivity. This flexibility allows for customization of the frost detection system to meet the specific requirements of different applications.

Future research should focus on enhancing the automation and intelligence of frost-sensing systems. This could involve the integration of machine learning algorithms or artificial intelligence techniques to improve the accuracy and reliability of frost detection and prediction. Through analyzing large datasets of sensor readings and operation conditions, advanced algorithms can learn patterns and optimize the timing and control of defrosting operations.

## Figures and Tables

**Table 1:** Selection of tiles for further analysis.

Tile	Selection Criteria	Objective of the Analysis
c1, g3	Tiles exhibiting the greatest variation in frost formation values (excluding those with artifacts such as fin damage and sensors).	Compare differences that can be observed within average tiles.
c2	Tile in which a resistive sensor was placed to monitor frost formation on the HX.	Determine the difference in accuracy between analyzing the overall average of the image (previous method) and analyzing a tile where the sensor is located. Compare the data from the tile, sensor, and the overall average of the image.
c4, f6, g5, a9	Tiles with artifacts such as fin damage and sensors.	Analyze the impact of defects such as a damaged fin or the obstruction caused by the temperature and humidity sensor on the readings of a small tile.

**Table 2:** Frost accumulation in the HX surface in five different points of the frosting phase and frost accumulation in selected tiles.

Frost formation	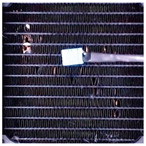	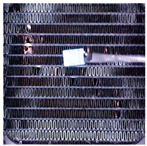	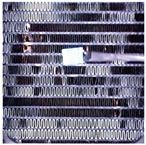	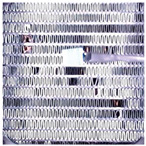	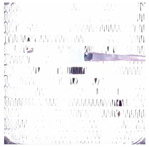
Tile g2	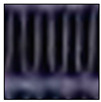	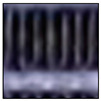	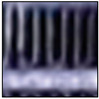	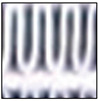	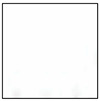
Tile d1	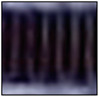	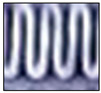	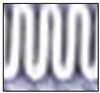	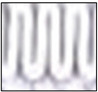	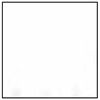
Tile g3	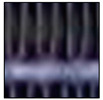	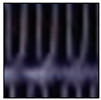	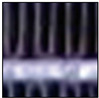	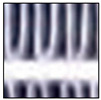	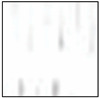

## Data Availability

Detailed tile data can be found through the link: https://drive.google.com/drive/folders/1BjBtqFqKxrIbLMyPedGwnqnYQ2Qztbps?usp=sharing.

## Nomenclature

*R*	Resistance (Ω)	*L*	Distance between electrodes (m)
*ρ*	resistivity (Ω.m)	*A*	Area (m^2^)
*B&W*	Black and White	*HX*	Heat exchanger
